# Evaluation of the content coverage of questionnaires containing basic and instrumental activities of daily living (ADL) used in adult patients with brain tumors

**DOI:** 10.1007/s11060-019-03136-9

**Published:** 2019-03-18

**Authors:** Quirien Oort, Martin J. B. Taphoorn, Sietske A. M. Sikkes, Bernard M. J. Uitdehaag, Jaap C. Reijneveld, Linda Dirven

**Affiliations:** 1 Department of Neurology and Brain Tumor Center Amsterdam, Amsterdam University Medical Centers (Location VUmc), PO BOX 7057, 1007 MB Amsterdam, The Netherlands; 2 Department of Neurology, Leiden University Medical Center, Leiden, The Netherlands; 3 Department of Neurology, Haaglanden Medical Center, The Hague, The Netherlands; 4 Department of Epidemiology and Biostatistics, Amsterdam University Medical Centers (Location VUmc), Amsterdam, The Netherlands; 5 Alzheimer Center, Amsterdam University Medical Centers (Location VUmc), Amsterdam, The Netherlands; 6 Department of Neurology, Amsterdam University Medical Centers (Location AMC), Amsterdam, The Netherlands

**Keywords:** ADL, Activities of daily living, Glioma, Brain tumor, Daily functioning

## Abstract

**Background:**

Everyday functioning can be assessed using measures of basic activities of daily living (BADL) or instrumental activities of daily living (IADL). The aim of this review was to provide an overview of the scope and specific content of BADL and/or IADL covered by currently used questionnaires in adult brain tumor patient studies.

**Methods:**

Electronic databases were searched up to April 2017 to identify all eligible questionnaires with items regarding BADL/IADL in studies with adult brain tumor patients. Articles were selected using predetermined in- and exclusion criteria. Items with similar content were clustered into domains based on type of activity.

**Results:**

Thirty-one unique questionnaires containing at least one BADL and/or IADL item were identified; 21 and 29 questionnaires containing ≥ 1 BADL or IADL item, respectively. The percentage of ADL items in these questionnaires ranged from 4 to 100%. Only two questionnaires were specifically developed to measure BADL (Barthel Index and Katz-ADL) and two specifically for IADL (Lawton-Brody IADL and preliminary IADL-BN). Content clustering revealed that IADL had a larger variation in content (31 domains, e.g. work or leisure time activities) compared to BADL (15 domains, e.g. mobility or bathing/washing).

**Conclusion:**

Thirty-one questionnaires previously used in brain tumor studies contained items on BADL and/or IADL and covered a wide range of content, in particular for IADL. It is currently unclear which BADL/IADL are most relevant for brain tumor patients, and this should therefore be evaluated. Next, existing questionnaires could be adapted or validated, or new measures can be developed to meet these needs.

**Electronic supplementary material:**

The online version of this article (10.1007/s11060-019-03136-9) contains supplementary material, which is available to authorized users.

## Introduction

Brain tumor patients exhibit a wide variety of symptoms and signs, which may have a negative impact on patients’ functioning and well-being. Both physical and cognitive deficits may cause a decline in a patient’s capability to perform activities of daily life, which may lead to decreased participation in society. Particularly for brain tumor patients, who have an incurable disease, maintenance of everyday functioning and well-being is at least as important as prolonged survival [1]. Measures of functioning and well-being have therefore become an important outcome in this patient population.

Everyday functioning is generally measured using “activities of daily living” (ADL) tools. ADL can be categorized into two subgroups; Basic Activities of Daily Living (BADL) and Instrumental Activities of Daily Living (IADL). BADL refers to the more basic tasks in everyday life, including self-maintenance skills such as bathing, dressing and toileting. IADL on the other hand, relies on more complex skills that require multiple cognitive processes, and include activities such as preparing a meal, participating in traffic, and doing finances [2, 3]. Whereas cognitive functioning to some extent is necessary for BADL, higher order cognitive skills are essential for IADL, such as problem solving, planning and flexibility of thinking. IADL are necessary to function autonomously within society, and because of their cognitive complexity they are prone to be affected by subtle cognitive deficits [4–6]. Measuring IADL in brain tumor patients is particularly valuable, as patients with primary [7] and metastatic brain tumors [8] often report cognitive deficits, which can therefore be expected to lead to interference in everyday functioning [9].

BADL and IADL are both useful measures in clinical research as well as in clinical practice. In clinical studies, BADL and IADL instruments may be included as secondary outcome measure, to quantify the impact of treatment on a patient’s functioning. In clinical practice, information on ADL can be used to monitor patients over time, or to evaluate the effects of neuro-rehabilitation. Although BADL have been assessed in brain tumor patients as part of neuro-rehabilitation practice [10–12], these are often generic outcome measures implemented for all types of patient groups. IADL, in contrast, is rarely systematically assessed in clinical practice. Moreover, both outcomes are rarely included in clinical trials for brain tumor patients, despite the fact they may provide important information on the patients’ functioning. Nevertheless, to improve ADL assessment in clinical trials or practice, several steps need to be taken. First, it needs to be established which instruments are already used for brain tumor patients and what content is covered by these instruments. A next step would be to determine which ADL domains are relevant for brain tumor patients, and to evaluate if the identified measures are appropriate for this purpose, or that existing measures need to be validated in brain tumor patients or that new measures should be developed.

The objective of this review was to provide an overview of the content coverage of all questionnaires containing BADL and/or IADL items that are currently used in studies with adult brain tumor patients. We specifically determined the number of instruments that included BADL and/or IADL items, the percentage of items in each instrument covering ADL, as well as the BADL and IADL domains that were covered.

## Methods

All procedures were according to the Preferred Reporting Items for Systematic Reviews and Meta-Analyses (PRISMA) guidelines [13].

### Data sources and search strategy

A literature review was conducted to identify all eligible questionnaires with items regarding ADL in studies with adult brain tumor patients, by searching the electronic databases PubMed, Embase, Cochrane, PsycINFO and CINAHL up to April 2017 (no lower limit of year). The search string consisted of a combination of three components, one related to brain tumors, one related to ADL, and one related to questionnaires (see Supplementary File for the complete search string in Pubmed).

### Selection criteria and process

Two reviewers (QO and LD) independently screened titles and abstracts for articles reporting the use of questionnaires possibly measuring ADL in adult brain tumor patients. Articles were deemed eligible if: they were original peer-reviewed articles (e.g. no reviews or conference abstracts), written in English, the patient population included at least 10 patients with glioma or brain metastases, patients were > 18 years, and if self-report or observer-reported questionnaires were used that contained at least one ADL item. Exclusion criteria were animal studies, studies including patients with childhood acquired brain tumors, or articles describing questionnaires without any BADL/IADL item (e.g. personality, mood or satisfaction/need questionnaires). After title/abstract screening, full-texts of potentially relevant articles were screened for eligibility applying the same criteria. Disagreements between reviewers were resolved by discussion until consensus was reached. Reference lists of included articles were reviewed for further eligible articles.

### Data extraction

For each eligible article, questionnaires were extracted and reviewed for potential ADL items. The same two reviewers determined whether items reflected ADL, according to the following definitions: BADL were defined as tasks that relate to the most basic self-maintenance skills that require lesser amounts of cognitive effort, while IADL were defined as complex, higher-order activities for which multiple cognitive processes are necessary [2]. Items were then selected based on the following criteria: items had to reflect (a) ADL, either BADL, IADL or containing both BADL and IADL in a single item, according to the predetermined definitions and (b) refer to the ability to perform the ADL (e.g., excluding items such as ‘I have work I like to do’). Disagreements between reviewers were resolved by discussion until consensus was reached. For each extracted questionnaire, the number and percentage of items considered as ADL, BADL, IADL, or items with both BADL and IADL in a single item, was evaluated. In addition, items were evaluated for their content. As this paper primarily aims to give a comprehensive overview of the content of ADL measures described in the current literature, no ADL outcome data was collected and therefore no factor analyses could be performed, but rather clustering of items with the same or very similar content, i.e. based on type of activity, as defined by two reviewers (QO and LD). Although subjectively clustered, a strict criterion was applied by giving each unique type of activity a separate content domain.

## Results

**Fig. 1:**
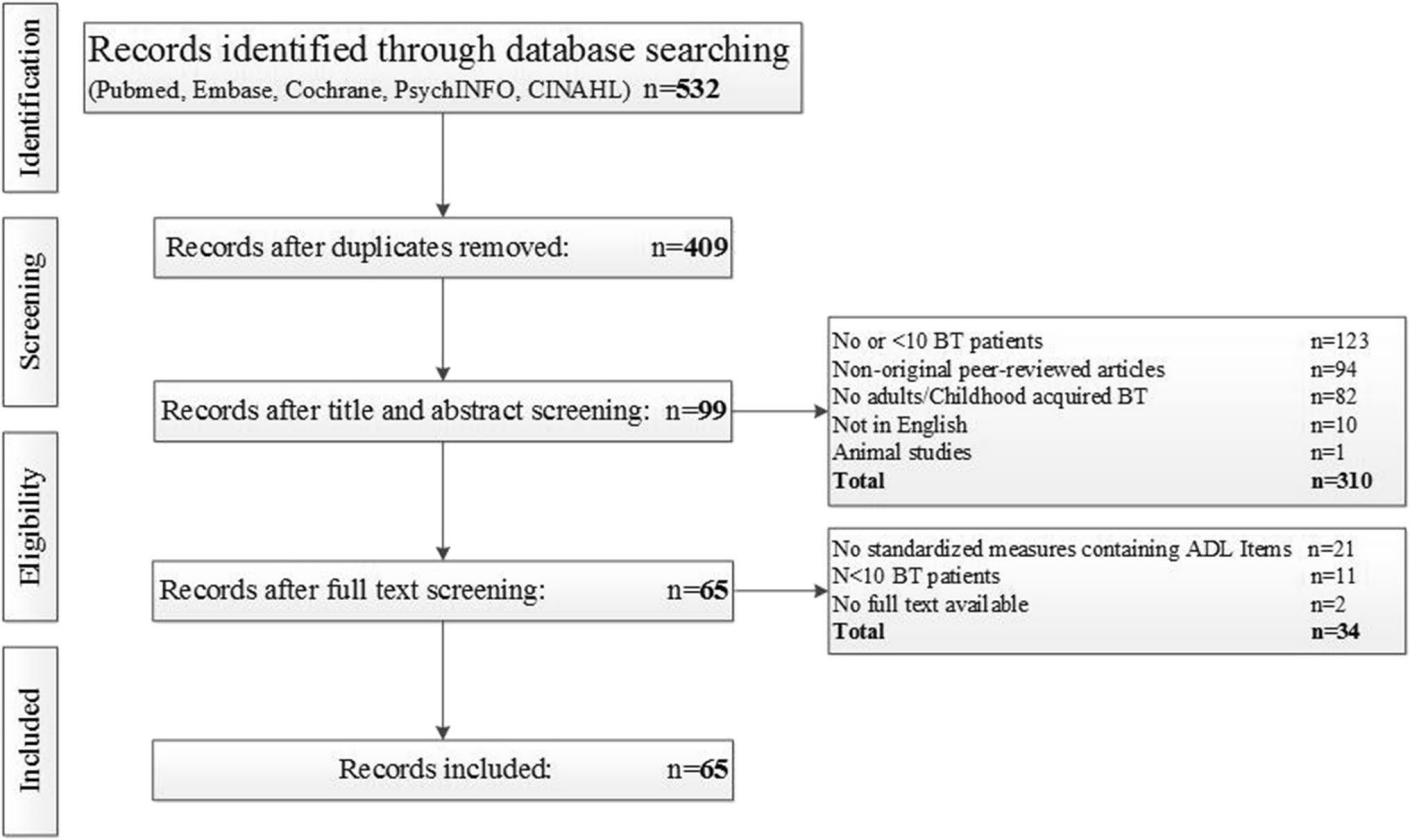
Flow diagram of record inclusion

We identified 532 records through database searching. After duplicates were removed, 409 unique records remained. Title and abstract screening excluded 310 records, leaving 99 records for full-text screening. Based on the same in- and exclusion criteria, another 34 records were excluded after reviewing the full-texts. In the remaining 65 articles, 31 unique questionnaires containing items on ADL were identified (see Fig. 1 for an overview of the screening procedure, and Table 1 for the questionnaires identified). No additional articles describing questionnaires were identified from reviewing the reference lists of the full-text articles.

**Table 1:** Outcome measures containing items on ADL

Outcome measures	Number (%) of articles using questionnaire	Number (%) of ADL; BADL, IADL or both items in questionnaire/subscale							
		ADL items							
		Total ADL		BADL		IADL		Both	
1. Barthel Index (BI)	28 (43%)	10/10	100%	10/10	100%	0/10	–	0/10	–
2. Functional independence measure (FIM)	16 (25%)	16/18	89%	13/18	72%	3/18	17%	0/18	–
Motor subscale		13/13	100%	13/13	100%	0/13	–	0/13	–
Cognitive subscale		3/5	60%	0/5	–	3/5	60%	0/5	–
3. EORTC Quality of Life Questionnaire Core 30 (EORTC QLQ-C30)	14 (22%)	8/30	27%	4/30	13%	4/30	13%	0/30	–
Global health status subscale		0/2	–	0/2	–	0/2	–	0/2	–
Physical function subscale		4/5	80%	4/5	80%	0/5	–	0/5	–
Role function subscale		2/2	100%	0/2	–	2/2	100%	0/2	–
Emotional function subscale		0/4	–	0/4	–	0/4	–	0/4	–
Cognitive function subscale		0/2	–	0/2	–	0/2	–	0/2	–
Social function subscale		2/2	100%	0/2	–	2/2	100%	0/2	–
Fatigue subscale		0/3	–	0/3	–	0/3	–	0/3	–
Nausea/vomiting subscale		0/2	–	0/2	–	0/2	–	0/2	–
Pain subscale		0/2	–	0/2	–	0/2	–	0/2	–
Single items		0/6	–	0/6	–	0/6	–	0/6	–
4. EORTC quality of life questionnaire brain module 20 (EORTC QLQ-BN20)	9 (14%)	2/20	10%	0/20	–	2/20	10%	0/20	–
Future uncertainty subscale		0/4	–	0/4	–	0/4	–	0/4	–
Visual disorder subscale		0/3	–	0/3	–	0/3	–	0/3	–
Motor dysfunction subscale		0/3	–	0/3	–	0/3	–	0/3	–
Communication deficit subscale		2/3	67%	0/3	–	2/3	67%	0/3	–
Single items		0/7	–	0/7	–	0/7	–	0/7	–
5. Functional assessment of cancer therapy-brain (FACT-Br)	8 (12%)	6/23	26%	1/23	4%	5/23	22%	0/23	–
6. Medical outcomes study 36-item short form survey (SF-36)	5 (8%)	15/36	42%	8/36	22%	5/36	14%	2/36	6%
Physical functioning subscale		10/10	100%	8/10	80%	0/10	–	2/10	20%
Role limitations due to physical health subscale		1/4	25%	0/4	–	1/4	25%	0/4	–
Role limitations due to emotional problems subscale		1/3	33%	0/3	–	1/3	33%	0/3	–
Energy/fatigue subscale		0/4	–	0/4	–	0/4	–	0/4	–
Emotional well-being subscale		0/5	–	0/5	–	0/5	–	0/5	–
Social functioning subscale		2/2	100%	0/2	–	2/2	100%	0/2	–
Pain subscale		1/2	50%	0/2	–	1/2	50%	0/2	–
General health subscale		0/5	–	0/5	–	0/5	–	0/5	–
Health change		0/1	–	0/1	–	0/1	–	0/1	–
7. Katz index of activities of daily living (Katz-ADL)	3 (5%)	6/6	100%	6/6	100%	0/6	–	0/6	–
8. Functional living index-cancer (FLIC)	3 (5%)	3/22	14%	0/22	–	3/22	14%	0/22	–
Physical subscale		3/7	43%	0/7	–	3/7	43%	0/7	–
Psychological subscale		0/6	–	0/6	–	0/6	–	0/6	–
Hardship subscale		0/5	–	0/5	–	0/5	–	0/5	–
Nausea subscale		0/2	–	0/2	–	0/2	–	0/2	–
Social subscale		0/2	–	0/2	–	0/2	–	0/2	–
9. Quality of life in epilepsy-31 inventory (QOLIE-31)	3 (5%)	6/31	19%	0/31	–	6/31	19%	0/31	–
Seizure worry subscale		0/5	–	0/5	–	0/5	–	0/5	–
Overall quality of life subscale		0/2	–	0/2	–	0/2	–	0/2	–
Emotional well-being subscale		0/5	–	0/5	–	0/5	–	0/5	–
Energy/fatigue subscale		0/4	–	0/4	–	0/4	–	0/4	–
Cognitive subscale		3/6	50%	0/6	–	3/6	50%	0/6	–
Medication effects subscale		0/3	–	0/3	–	0/3	–	0/3	–
Social function subscale		3/5	60%	0/5	–	3/5	60%	0/5	–
General health subscale		0/1	–	0/1	–	0/1	–	0/1	–
10. Brief fatigue inventory (BFI) [Question 4]	3 (5%)	2/6	33%	1/6	17%	1/6	17%	0/6	–
11. Spitzer quality of life index (SQLI)	2 (3%)	2/5	40%	0/5	–	1/5	20%	1/5	20%
12. Perceived impact of problem profile (PIPP)	2 (3%)	15/23	65%	8/23	35%	7/23	30%	0/23	–
Self-care subscale		4/4	100%	4/4	100%	0/4	–	0/4	–
Mobility subscale		5/5	100%	4/5	80%	1/5	20%	0/5	–
Participation subscale		5/5	100%	0/5	–	5/5	100%	0/5	–
Relationships subscale		0/4	–	0/4	–	0/4	–	0/4	–
Psychological well-being subscale		1/5	20%	0/5	–	1/5	20%	0/5	–
13. CAncer rehabilitation evaluation system-short form (CARES-SF)	2 (3%)	10/59	17%	3/59	5%	7/59	12%	0/59	–
Physical subscale		5/10	50%	2/10	20%	3/10	30%	0/10	–
Psychosocial subscale		1/17	6%	0/17	–	1/17	6%	0/17	–
Medical interaction subscale		0/4	–	0/4	–	0/4	–	0/4	–
Marital subscale		0/6	–	0/6	–	0/6	–	0/6	–
Sexual subscale		1/3	33%	1/3	33%	0/3	–	0/3	–
Miscellaneous subscale		3/19	16%	0/19	–	3/19	16%	0/19	–
14. Brain Symptom and Impact Questionnaire (BASIQ)	2 (3%)	6/18	33%	3/18	17%	3/18	17%	0/18%	–
15. Community Integration Questionnaire (CIQ)	2 (3%)	13/15	87%	0/15	–	13/15	87%	0/15	–
Home integration subscale		5/5	100%	0/5	–	5/5	100%	0/5	–
Social integration subscale		4/6	67%	0/6	–	4/6	67%	0/6	–
Productivity subscale		4/4	100%	0/4	–	4/4	100%	0/4	–
16. Functional assessment measure (FAM)	1 (2%)	4/12	33%	1/12	8%	3/12	25%	0/12	–
Motor subscale		2/3	67%	1/3	33%	1/3	33%	0/3	–
Cognitive subscale		2/9	22%	0/9	–	2/9	22%	0/9	–
17. Functional assessment of cancer therapy-general (FACT-G)	1 (2%)	1/27	4%	0/27	–	1/27	4%	0/27	–
Physical well-being subscale		0/7	–	0/7	–	0/7	–	0/7	–
Social/family well-being subscale		0/7	–	0/7	–	0/7	–	0/7	–
Emotional well-being subscale		0/6	–	0/6	–	0/6	–	0/6	–
Functional well-being subscale		1/7	14%	0/7	–	1/7	14%	0/7	–
18. Lawton-Brody Instrumental activities of daily living (Lawton-Brody iADL)	1 (2%)	8/8	100%	0/8	–	8/8	100%	0/8	–
19. Cognitive Functioning Subscale of the Medical Outcomes Scale (MOS CFS)	1 (2%)	4/6	67%	0/6	–	4/6	67%	0/6	–
20. 15D	1 (2%)	4/15	27%	3/15	20%	1/15	7%	0/15	–
21. Brief pain inventory (BPI)	1 (2%)	2/7	29%	1/7	14%	1/7	14%	0/7	–
22. Disability rating scale (DRS)	1 (2%)	4/8	50%	3/8	38%	1/8	13%	0/8	–
23. International classification of functioning, disability and health (ICF)	1 (2%)								
Part 2: Activity limitations & participation restriction		30/48	63%	9/48	19%	20/48	42%	1/48	2%
Learning and applying knowledge subscale		6/6	100%	0/6	–	6/6	100%	0/6	–
General tasks and demands subscale		1/2	50%	0/2	–	1/2	50%	1/2	50%
Communication subscale		3/5	60%	0/5	–	3/5	60%	0/5	–
Mobility subscale		5/6	83%	3/6	50%	2/6	33%	0/6	–
Self-care subscale		7/7	100%	6/7	86%	1/7	14%	0/7	–
Domestic life subscale		4/4	100%	0/4	–	4/4	100%	0/4	–
Interpersonal interactions and relationships subscale		0/7	–	0/7	–	0/7	–	0/7	–
Major life areas subscale		1/6	17%	0/6	–	1/6	17%	0/6	–
Community, social and civic life subscale		2/5	40%	0/5	–	2/5	40%	0/5	–
24. Nottingham extended activities of daily living (NEADL)	1 (2%)	22/22	100%	6/22	27%	16/22	73%	0/22	–
Mobility subscale		6/6	100%	4/6	67%	2/6	33%	0/6	–
Kitchen subscale		5/5	100%	2/5	40%	3/5	60%	0/5	–
Domestic subscale		5/5	100%	0/5	–	5/5	100%	0/5	–
Leisure subscale		6/6	100%	0/6	–	6/6	100%	0/6	–
25. Nottingham health profile (NHP)	1 (2%)	11/45	24%	6/45	13%	5/45	13%	0/45	–
Part 1		5/38	13%	5/38	13%	0/38	–	0/38	–
Physical mobility subscale		5/8	63%	5/8	63%	0/8	–	0/8	–
Pain subscale		0/8	–	0/8	–	0/8	–	0/8	–
Sleep subscale		0/5	–	0/5	–	0/5	–	0/5	–
Energy subscale		0/3	–	0/3	–	0/3	–	0/3	–
Social isolation subscale		0/5	–	0/5	–	0/5	–	0/5	–
Emotional reactions subscale		0/9	–	0/9	–	0/9	–	0/9	–
Part 2. Seven life areas affected		6/7	86%	1/7	14%	5/7	71%	0/7	–
Work		1		–		1		–	
Looking after the home		1		–		1		–	
Social life		1		–		1		–	
Home life		–		–		–		–	
Sex life		1		1		–		–	
Interests and hobbies		1		–		1		–	
Vacations		1		–		1		–	
26. Rotterdam symptom checklist (RSCL)	1 (2%)	8/38	21%	4/38	11%	4/38	11%	0/38	–
Activity level subscale		8/8	100%	4/8	50%	4/8	50%	0/8	–
Single items		0/30	–	0/30	–	0/30	–	0/30	–
27. Piper Fatigue Scale (PFS)	1 (2%)	4/27	15%	1/27	4%	3/27	11%	0/27	–
Behavioral/severity subscale		4/6	67%	1/6	17%	3/6	50%	0/6	–
Affective meaning subscale		0/5	–	0/5	–	0/5	–	0/5	–
Sensory subscale		0/5	–	0/5	–	0/5	–	0/5	–
Cognitive/mood subscale		0/6	–	0/6	–	0/6	–	0/6	–
Single items		0/5	–	0/5	–	0/5	–	0/5	–
28. Preliminary IADL item list for brain tumor patients (IADL-BN)	1 (2%)	32/32	100%	0/32	–	32/32	100%	0/32	–
Household activities subscale		4/4	100%	0/4	–	4/4	100%	0/4	–
Finances and administration subscale		5/5	100%	0/5	–	5/5	100%	0/5	–
Appliances subscale		4/4	100%	0/4	–	4/4	100%	0/4	–
Work subscale		2/2	100%	0/2	–	2/2	100%	0/2	–
Appointments subscale		3/3	100%	0/3	–	3/3	100%	0/3	–
Social activities subscale		2/2	100%	0/2	–	2/2	100%	0/2	–
Transport subscale		4/4	100%	0/4	–	4/4	100%	0/4	–
Leisure subscale		2/2	100%	0/2	–	2/2	100%	0/2	–
General subscale		6/6	100%	0/6	–	6/6	100%	0/6	–
29. Self-administered 10-point Likert self-assessment quality of life scale	1 (2%)	3/16	19%	1/16	6%	2/16	13%	0/16	–
30. Self-perceived deficits in attention (FEDA)	1 (2%)	15/27	52%	1/27	4%	13/27	48%	1/27	7%
Distractibility and retardation in mental processes subscale		7/13	54%	1/13	8%	5/13	38%	1/13	8%
Fatigue and retardation in activities of daily living subscale		5/8	63%	0/8	–	5/8	63%	0/8	–
Decrease in drive subscale		3/6	50%	0/6	–	3/6	50%	0/6	–
31. Sydney Psychosocial Reintegration Scale (SPRS)	1 (2%)	4/12	33%	0/12	–	4/12	33%	0/12	–
Part A. Work and leisure		3/4	75%	0/4	–	3/4	75%	0/4	–
Part B. Interpersonal relationships subscale		0/4	–	0/4	–	0/4	–	0/4	–
Part C. Living skills subscale		1/4	25%	0/4	–	1/4	25%	0/4	–

The 31 identified questionnaires included a total of 672 items. These 672 items were reviewed, and items considered measuring BADL or IADL according to the predefined criteria were extracted. In 94.6% of cases, the reviewers agreed on categorizing the items as BADL, IADL, both within a single item or neither. There were 21 (68%) questionnaires containing at least one BADL item and 29 (94%) questionnaires containing at least one IADL item. The percentage of ADL items in these questionnaires ranged from 4%-100%; between 0%-100% for both BADL and IADL items (see Table 1). Twelve (38%) questionnaires had at least 50% of items on ADL of which three (10%) questionnaires at least 50% of items on BADL and six (19%) at least 50% of items on IADL (Table 1).

The clustering of items into domains resulted in a total of 15 domains for BADL and 31 domains for IADL (Tables 2, 3, respectively). In addition, some items could be considered both BADL and IADL, such as the item ‘undertaking a single task’, depending on the complexity of the task. In accordance with the American Occupational Therapy Association [14], ‘sexual activity’, an activity used in four of the questionnaires, was considered not to be a higher order cognitively complex activity, and therefore classified as BADL.

**Table 2:** BADL domains extracted from outcome measures

Activity	Included in # of questionnaires	Questionnaire abbreviations
Mobility	13	BI, FIM, EORTC QLQ-C30, ICF, SF-36, BFI, PIPP, BASIQ, BPI, RSCL, 15D, NHP, NEADL
Bathing/washing^a^	9	BI, KATZ-ADL, FIM, ICF, BASIQ, SF-36, PIPP, CARES-SF, FEDA
Dressing^a^	8	BI, KATZ-ADL, FIM, ICF, BASIQ, SF-36, PIPP, NHP
Feeding/eating/drinking^a^	8	BI, KATZ-ADL, FIM, ICF, PIPP, DRS, 15D, NEADL
Stairs	7	BI, FIM, SF-36, RSCL, 15D, NHP, NEADL
Toilet use^a^	6	BI, KATZ-ADL, FIM, ICF, PIPP, DRS
Self-care in general	6	EORTC QLQ-C30, FACT-Br, SQLI, RSCL, Self-adm. 10-Point Likert self-assessment QOL, FEDA
Grooming^a^	5	BI, FIM, ICF, CARES-SF, DRS
Transferring (bed/chair/wheelchair/toilet/tub/shower/car)	5	BI, KATZ-ADL, FIM, FAM, NEADL
Lifting/carrying/moving objects	5	EORTC QLQ-C30, ICF, SF-36, PIPP, CARES-SF
Sexual activity	4	CARES-SF, PFS, 15D, NHP
Passive mobility (sitting, standing, bending etc.)	3	SF-36, PIPP, NHP
Bladder management	3	BI, KATZ-ADL, FIM
Bowel management	3	BI, KATZ-ADL, FIM
Undertaking a single task^b^	1	ICF

*EORTC QLQ-C30* EORTC Quality of Life Questionnaire Core 30, *FACT-Br* functional assessment of cancer therapy-brain, *SQLI* Spitzer Quality of Life Index, *ICF* International classification of functioning, disability and health, *PIPP* perceived impact of problem profile, *BASIQ* brain symptom and impact questionnaire, *RSCL* Rotterdam Symptom Checklist, *FEDA* self-perceived deficits in attention, *BI* Barthel Index, *Katz-ADL* Katz index of activities of daily living, *FIM* functional independence measure, *DRS* disability rating scale, *NEADL* Nottingham extended activities of daily living, *SF-36* Medical Outcomes Study 36-Item Short Form Survey, *CARES-SF* CAncer Rehabilitation Evaluation System-Short Form, *NHP* Nottingham health profile, *FAM* functional assessment measure, *BFI* brief fatigue inventory, *BPI* brief pain inventory, *PFS* Piper Fatigue Scale

^a^Mentioned as separated item

^b^Depending on the difficulty of the tasks, an item could be classified as BADL or IADL

**Table 3:** IADL domains extracted from outcome measures

Activity	Included in # of questionnaires	Questionnaire abbreviations
Work (also studying/volunteering/homemaking)	18	Preliminary IADL-BN, NEADL, PIPP, CARES-SF, SF-36, NHP, FACT-G, EORTC QLQ-C30, RSCL, SPRS, PFS, SQLI, Self-adm. 10-Point Likert self-assessment QOL, 15D, DRS, BFI, BPI, CIQ
Housekeeping/chores^a^	12	Lawton-Brody IADL, preliminary IADL-BN, NEADL, CARES-SF, ICF, NHP, RSCL, FLIC, BASIQ, Self-adm. 10-Point Likert self-assessment QOL, 15D, CIQ
Social activities	12	NEADL, CARES-SF, PIPP, ICF, QOLIE-31, SF-36, NHP, FIM, EORTC QLQ-C30, PFS, Self-adm. 10-Point Likert self-assessment QOL, CIQ
Leisure time (hobby’s, sports, vacation)	12	FEDA, PIPP, ICF, QOLIE-31, SF-36, NHP, EORTC QLQ-C30, SPRS, FLIC, PFS, 15D, CIQ
Use of transport/travel around	10	Lawton-Brody IADL, preliminary IADL-BN, NEADL, PIPP, CARES-SF, ICF, FAM, SPRS, SQLI, CIQ
Communicating/expressing	8	Preliminary IADL-BN, CARES-SF, FIM, FEDA, ICF, FACT-Br, EORTC QLQ-BN20, BASIQ
Reading (book/newspaper/magazine/manuals)	8	Preliminary IADL-BN, NEADL, FEDA, ICF, QOLIE-31, FAM, FACT-Br, BASIQ
Preparing a meal^a^	6	Lawton-Brody IADL, preliminary IADL-BN, NEADL, ICF, FLIC, CIQ
Shopping (grocery, clothing or other products)	6	Lawton-Brody IADL, preliminary IADL-BN, NEADL, ICF, RSCL, CIQ
Finances and administration (handling money, filling in forms)	6	Lawton-Brody IADL, preliminary IADL-BN, NEADL, FEDA, ICF, CIQ
General task related (taking longer, not able to start, not able to finish, getting distracted, unable to focus on next task, difficulties overseeing the sequence of steps for completion, not able to adapt to unexpected changes)	6	Preliminary IADL-BN, FEDA, ICF, QOLIE-31, SF-36, MOS CFS
Driving a car^a^	5	Preliminary IADL-BN, NEADL, ICF, QOLIE-31, FACT-Br
Writing	4	NEADL, ICF, FAM, FACT-Br
Reasoning and solving problems	4	ICF, FIM, QOLIE-31, MOS CFS
Appointments^a^ (making and keeping appointments, planning and organising the activity)	4	Preliminary IADL-BN, CARES-SF, SPRS, MOS CFS
Family life/caring for family members	3	PIPP, EORTC QLQ-C30, CIQ
‘Modern’ appliances ((mobile) phone, computer, laptop, tablet, Satnav)	3	Lawton-Brody IADL, preliminary EORTC IADL-BN, NEADL
Orientating in traffic (crossing road/cope with road traffic/finding the way)	3	Preliminary IADL-BN, NEADL, FEDA
Managing own medication	2	Lawton-Brody IADL, preliminary EORTC IADL-BN
Watching/comprehending television programs or movies	2	Preliminary IADL-BN, FEDA
Keeping track were you put things	2	Preliminary IADL-BN, MOS CFS
Able to live independently	2	PIPP, CARES-SF
Undertaking multiple tasks/multitasking	2	Preliminary IADL-BN, ICF
Undertaking a single task^b^	1	ICF
Calculating	1	ICF
Learning new things^a^	1	Preliminary IADL-BN
Ability to use household appliances	1	Preliminary EIADL-BN
Making decisions^a^	1	FACT-Br
Assisting others	1	ICF
Watching^c^	1	ICF
Listening^c^	1	ICF

^a^Mentioned as separated item

^b^Depending on the difficulty of the tasks, it could be classified as BADL or IADL

^c^Looking and hearing was deemed passive (e.g. BADL), watching and listening was deemed active and engaging (e.g. IADL)

### ADL specific questionnaires

A total of five questionnaires were specifically developed to measure ADL; one measuring ADL in general, two focusing on BADL specifically and two focusing on IADL. The remaining questionnaires were not primarily designed to measure ADL, but for example health-related quality of life.

The Nottingham Extended Activities of Daily Living (NEADL) [15] was specifically developed to measure ADL in general, with 6/22 (27%) items BADL, and the remaining 16/22 (73%) items that were considered IADL.

The two instruments measuring BADL were the Barthel Index (BI) [16] and the Katz Index of Activities of Daily Living (Katz-ADL) [17]. The BI (or a modified BI) was the most commonly used instrument, included in 43% of the studies. It is a 10-item outcome measure that is completed by a health care professional. All 10 items of the BI were considered to measure BADL. The Katz-ADL is a 6-item measurement that also has to be completed by a health care professional, and includes items that are similar to the BI. This questionnaire however, was only used in 5% of the studies. All 6 items were considered to measure BADL.

The two questionnaires specifically developed (or in the processes of being developed) to measure IADL were the Lawton-Brody Instrumental Activities of Daily Living (Lawton-Brody IADL) [18] and the preliminary IADL item list for brain tumor patients (preliminary IADL-BN) [19]. Both questionnaires were used in only 2% of the studies. The Lawton-Brody IADL, to be completed by a health care professional, consists of eight items, which were all considered to be IADL. Likewise, all 32 items in the preliminary IADL-BN [19] were considered to reflect IADL. For this questionnaire, both patient-based and proxy-based versions are available.

### Questionnaires with items on basic activities of daily living

Besides the two abovementioned BADL specific questionnaires (BI and Katz-ADL), there were 19 other questionnaires with BADL items. The Functional Independence Measure (FIM) [20] was the only questionnaire not specifically developed to measure ADL that contained ≥ 50% items referring to BADL. It is a global measure of independence and has two subscales, the Motor and Cognitive subscale. The FIM comprises 13/18 (72%) BADL items, all from the Motor subscale (13 items).

Seven other questionnaires had only subscales with ≥ 50% of the items referring to BADL (Table 1). Six out of these seven questionnaires had subscales on physical/mobility with 50–80% of the items referring to BADL. Two out of seven questionnaires had subscales on self-care with 86–100% of the items referring to BADL. The only self-care subscale item not considered BADL was an International Classification of Functioning, disability and health (ICF) [21] item which was considered to represent IADL instead of BADL, because ‘looking after one’s health’ was deemed to require higher order cognitive skills to perform. Furthermore, the Rotterdam Symptom Checklist (RSCL) [22] has an activity level subscale with ≥ 50% of the items on BADL.

Eleven other questionnaires had only a few items containing BADL (1–3 items; 4–38% of the questionnaire), mostly either related to self-care activities, mobility or sexual activities.

### Questionnaires with items on instrumental activities of daily living

Besides the two IADL specific questionnaires (Lawton-Brody IADL and preliminary IADL-BN) and the NEADL, there were three other questionnaires not specifically developed to measure ADL were identified in which ≥ 50% items were considered IADL (Table 1). The Community Integration Questionnaire (CIQ) [23], a measurement of community integration, had 13/15 (87%) items reflecting IADL. The Functional Assessment Measure (FAM) [24], which is an expansion of the Functional Independence Measure (FIM) measuring independence, had 6/12 (50%) items reflecting IADL. Lastly, the Cognitive Functioning Subscale of the Medical Outcomes Scale (MOS CFS) [25] (4/6 items reflecting IADL, 67%) measures impairment across a range of cognitive functions.

Eleven other questionnaires had only subscales containing ≥ 50% items that could be considered IADL. Common subscales with ≥ 50% items reflecting IADL were social functioning (n = 3; 60–100% of the subscale), communication (n = 2; 60–67% of the subscale), and cognition (n = 2; 50–60% of the subscale). The remaining 12 questionnaires comprised only a few IADL items (1–9 items; 4–40% of the questionnaire), with no subscales with ≥ 50% items were considered IADL.

### Content coverage

When reviewing the content of the items considered BADL and IADL, items with same or very similar content (i.e. type of activity) were categorized. Unsurprisingly, the most common BADL domains were ‘mobility’ and ‘self-care’ (Tables 2, 3). Thirteen of the 21 questionnaires (62%) with BADL items included items on ‘mobility’. All but four questionnaires with BADL items had items regarding self-care (n = 18; 82%), either in ‘general’ (27%) and/or measured more specifically as items on ‘bathing/washing’ (41%), ‘feeding/eating/drinking’ (36%), ‘dressing’ (36%), ‘toilet use’ (27%) and ‘grooming’ (23%). The most common IADL domain was ‘work’ (18/29; 62%), comprising items referring to either work, employability, homemaking, studying or volunteering. Other common items were clustered as ‘housekeeping/chores’ (41%), ‘social activities’ (41%), ‘leisure time activities’ (41%) and ‘use of transport’ (34%). See Tables 2 and 3 for all BADL and IADL domains.

Table 4 shows the number and percentage of content domains covered by each of the 31 questionnaires. The BADL questionnaires BI and Katz-ADL cover 10/15 (67%) BADL domains and 6/15 (40%) domains, respectively. The Lawton-Brody IADL contains eight IADL items covering 7/31 (23%) domains, with two items falling under the domain ‘housekeeping/chores’ (i.e. items on housekeeping and laundry). The preliminary IADL-BN measure is currently further developed, but the pilot version contained 32 IADL items covering 19/31 (61%) domains, including the otherwise not covered domains’learning new things’ and ‘ability to use household appliances’. Although not developed to measure BADL specifically, other questionnaires also covered more than half of the domains, such as the FIM (+ FAM) (67%) and ICF (53%). The ICF also covers 19/31 (61%) IADL domains. The NEADL (6/22 BADL and 16/22 IADL items) covered 4/15 (27%) BADL domains and 12/31 (32%) IADL domains. Other questionnaires had content coverage ranging from 0 to 46%.

**Table 4:** Outcome measures content coverage

Outcome measures	Number (%) BADL and IADL domains			
	BADL		IADL	
Barthel Index (BI)	10/15	67%	0/31	–
Katz Index of Activities of Daily Living (Katz-ADL)	6/15	40%	0/31	–
Preliminary IADL item list for brain tumor patients (IADL-BN)	0/15	–	19/31	61%
Lawton-Brody Instrumental Activities of Daily Living (Lawton-Brody iADL)	0/15	–	7/31	23%
Nottingham extended activities of daily living (NEADL)	4/15	27%	12/31	39%
International Classification of Functioning, disability and health (ICF)	8/15	53%	19/31	61%
Functional Assessment Measure (FIM/FAM)	10/15	67%	6/31	19%
Perceived Impact of Problem Profile (PIPP)	7/15	47%	6/31	19%
Functional Independence Measure (FIM)	8/15	53%	3/31	10%
CAncer Rehabilitation Evaluation System-Short Form (CARES-SF)	4/15	27%	7/31	23%
Medical Outcomes Study 36-Item Short Form Survey (SF-36)	6/15	40%	4/31	13%
Community Integration Questionnaire (CIQ)	0/15	–	9/31	29%
Self-perceived deficits in attention (FEDA)	2/15	13%	7/31	23%
Nottingham Health Profile (NHP)	5/15	33%	4/31	13%
EORTC Quality of Life Questionnaire Brain module 20 (EORTC QLQ-C30/BN20)	3/15	20%	5/31	16%
Functional Assessment of Cancer Therapy-Brain (FACT-G/Br)	1/15	7%	6/31	19%
EORTC Quality of Life Questionnaire Core 30 (EORTC QLQ-C30)	3/15	20%	4/31	13%
15D	4/15	27%	3/31	10%
Quality of life in epilepsy-31 inventory (QOLIE-31)	0/15	–	6/31	19%
Brain Symptom and Impact Questionnaire (BASIQ)	3/15	20%	3/31	10%
Rotterdam Symptom Checklist (RSCL)	3/15	20%	3/31	10%
Cognitive Functioning Subscale of the Medical Outcomes Scale (MOS CFS)	0/15	–	4/31	13%
Sydney Psychosocial Reintegration Scale (SPRS)	0/15	–	4/31	13%
Disability Rating Scale (DRS)	3/15	20%	1/31	3%
Piper Fatigue Scale (PFS)	1/15	7%	3/31	10%
Self-administered 10-point Likert self-assessment quality of life scale	1/15	7%	3/31	10%
Functional Living Index-Cancer (FLIC)	0/15	–	3/31	10%
Spitzer Quality of Life Index (SQLI)	1/15	7%	2/31	7%
Brief Fatigue Inventory (BFI) [Question 4]	1/15	7%	1/31	3%
Brief Pain Inventory (BPI)	1/15	7%	1/31	3%
Functional Assessment of Cancer Therapy-General (FACT-G)	0/15	–	1/31	3%

*Lawton-Brody iADL* Lawton-Brody instrumental activities of daily living, *IADL-BN* preliminary IADL item list for brain tumor patients, *NEADL* Nottingham extended activities of daily living, *FEDA* Self-perceived deficits in attention, *CARES-SF* CAncer rehabilitation evaluation system-short form, *ICF* International classification of functioning, disability and health, *SF-36* medical outcomes study 36-item short form survey, *NHP* Nottingham health profile, *RSCL* Rotterdam symptom checklist, *FLIC* functional living index-cancer, *BASIQ* brain symptom and impact questionnaire, *SQLI* spitzer quality of life index, *DRS* disability rating scale, *BFI* brief fatigue inventory, *BPI* brief pain inventory, *CIQ* community integration questionnaire, *PIPP* perceived impact of problem profile, *QOLIE-31* quality of life in epilepsy-31 inventory, *FAM* functional assessment measure, *FACT-Br* functional assessment of cancer therapy-brain, *SPRS* Sydney psychosocial reintegration scale, *FACT-G* functional assessment of cancer therapy-general, *PFS* piper fatigue scale, *MOS CFS* cognitive functioning subscale of the medical outcomes scale, *EORTC QLQ-C30* EORTC quality of life questionnaire Core 30, *EORTC QLQ-BN20* EORTC quality of life questionnaire brain module 20

## Discussion

Thirty-one unique self-report or observer-reported questionnaires with items regarding ADL were identified in this systematic literature review. The majority (68%) of questionnaires had ≥ 1 item on BADL, almost all questionnaires (94%) had ≥ 1 item on IADL, and more than half (58%) of the questionnaires contained items on both BADL and IADL. Fifteen BADL (e.g. mobility and washing/dressing) and thirty-one IADL domains (e.g. work and housekeeping/chores) were identified, some addressed by a single questionnaire and others by up to eighteen questionnaires.

Although BADL and IADL are very useful outcome measures in both clinical practice and clinical trials, instruments addressing solely BADL/IADL which are specifically developed for and validated in adult brain tumor patients are currently lacking. Nevertheless, many used instruments contained some BADL (4–61%) or IADL (4–87%) items, providing information on ADL functioning. Whether these items are actually relevant for brain tumor patients remains to be investigated. Indeed, to accurately measure BADL and IADL in brain tumor patients, measures should be available that are fully relevant for the patient population and have good psychometric properties. Whereas the BI and Katz-ADL were specifically developed to measure BADL, and the Lawton-Brody for IADL, these questionnaires are not yet psychometrically validated in brain tumor patients. Validation of existing ADL scales is particularly important for this patient group given the considerable complex relation between the abilities to perform ADLs and the diversity in brain tumor characteristics (e.g. tumor location, tumor grade, tumor growth rate). The ADL scale must be valid and reliable for all brain tumor types and stages for it to be an accurate measure. This entails having a scale with good content validity, besides other psychometric properties.

The BI was originally developed to assess the change in functional status in individuals with neurologic or musculoskeletal disorders undergoing neurorehabilitation [16] and is among the most commonly used measures of functional status [26]. The BI has been shown to be reliable and valid in neurorehabilitation patient groups such as stroke and hospitalized patients, and the elderly [27–31]. For the Katz-ADL on the other hand, although implemented regularly in neurorehabilitation and research studies (mainly in elderly patients and neurorehabilitation patients [32–34]), very little evidence exists for its validity and reliability [26]. The BI seems to be the most promising BADL scale for adult patients with brain tumors, as it covers a large amount of different BADL domains. However, a validation is needed to assess if the BI adequately measures BADL in adult brain tumor patients, regardless of tumor characteristics, particularly with respect to the domains included. In case not all relevant domains are included, it should be considered to develop a brain tumor specific BI. The Lawton-Brody IADL questionnaire is commonly used in studies with patients with neurological problems, such as dementia [35], stroke [36] and traumatic brain injury [37]. However, it remains to be investigated if this questionnaire covers the full construct of IADL relevant for brain tumor patients. A pilot study evaluating the applicability of a dementia-specific IADL questionnaire for brain tumor patients showed that this particular questionnaire was only partly applicable to glioma patients, and that the addition of glioma-specific IADL activities is necessary to capture the IADL construct for this patient population [19]. Therefore, the EORTC IADL-BN questionnaire is under development, to specifically measure IADL that are relevant for brain tumor patients. This questionnaire is being developed to accurately measure IADL in brain tumor patients, irrespective of patient- and tumor-related characteristics and received treatments.

The remaining questionnaires we identified in this study either had a limited number of items on BADL or IADL, were not psychometrically validated in brain tumor patients, or both. These results underline that many questionnaires are used in brain tumor research that are possibly not suitable for assessing BADL or IADL in this patient population. It may therefore be questioned if appropriate conclusions can be drawn on BADL and IADL functioning in brain tumor patients with the current outcome measures. Studies validating existing questionnaires in brain tumor patients, or aiming to develop new instruments, therefore seem warranted.

A potential limitation of this review might be that certain questionnaires, and therefore certain items containing BADL or IADL, were missed due to the search strategy that was applied. Another limitation is that the classification of items into BADL and IADL was suboptimal, as the classification process is based on the judgement of the reviewers and based on a definition that may not perfectly reflect the underlying constructs of these concepts. Nevertheless, the reviewers did agree on the classification in 94.6% of cases, suggesting that the classification was quite straight-forward. Some activities, however, were subject to extensive discussion as these could be perceived as BADL and/or IADL (e.g. sexual activity). In those cases, the literature was used to classify activities. Moreover, the domains were composed based on similar content, as determined by the authors, which may not overlap with domains as mentioned in other studies.

In conclusion, 31 unique questionnaires previously used in adult brain tumor studies included items on BADL and/or IADL, covering a wide range of content, particularly for IADL. Whether this content addresses all underlying aspects of the construct of BADL and IADL that are relevant for brain tumor patients remains to be determined. Subsequently, existing questionnaires could be validated to accurately measure the full constructs of BADL and IADL in brain tumor patients, or new measures can be developed. Adequate measurement of BADL and IADL may be accomplished with a full psychometric validation of the BI in the brain tumor population and the development of the EORTC IADL-BN questionnaire specifically for brain tumor patients, respectively.

## Electronic supplementary material

Below is the link to the electronic supplementary material.

Supplementary material 1 (DOCX 14 KB)

## References

[1] Efficace F, Taphoorn M (2012). Methodological issues in designing and reporting health-related quality of life in cancer clinical trials: the challenge of brain cancer studies. J Neurooncol.

[2] Sikkes SA (2012). A new informant-based questionnaire for instrumental activities of daily living in dementia. Alzheimers Dement.

[3] Sikkes SA (2009). A systematic review of instrumental activities of daily living scales in dementia: room for improvement. J Neurol Neurosurg Psychiatry.

[4] Ahn IS (2009). Impairment of instrumental activities of daily living in patients with mild cognitive impairment. Psychiatry Investig.

[5] Jekel K (2015). Mild cognitive impairment and deficits in instrumental activities of daily living: a systematic review. Alzheimers Res Ther.

[6] Reppermund S (2011). The relationship of neuropsychological function to instrumental activities of daily living in mild cognitive impairment. Int J Geriatr Psychiatry.

[7] van Kessel E (2017). Tumor-related neurocognitive dysfunction in patients with diffuse glioma: a systematic review of neurocognitive functioning prior to anti-tumor treatment. J Neurooncol.

[8] Habets EJ (2016). Neurocognitive functioning and health-related quality of life in patients treated with stereotactic radiotherapy for brain metastases: a prospective study. Neuro Oncol.

[9] Godbout L (2005). Cognitive structure of executive deficits in patients with frontal lesions performing activities of daily living. Brain Inj.

[10] Pace A (2007). Home rehabilitation for brain tumor patients. J Exp Clin Cancer Res.

[11] Kim BR (2012). Fatigue assessment and rehabilitation outcomes in patients with brain tumors. Support Care Cancer.

[12] Han EY (2015). Functional improvement after 4-week rehabilitation therapy and effects of attention deficit in brain tumor patients: comparison with subacute stroke patients. Ann Rehabil Med.

[13] Moher D (2009). Preferred reporting items for systematic reviews and meta-analyses: the PRISMA statement. PLoS Med.

[14] Roley SS (2008). Occupational therapy practice framework: domain & practice. Am J Occup Ther.

[15] Lincoln NB, Gladman JR (1992). The extended activities of daily living scale: a further validation. Disabil Rehabil.

[16] Mahoney FI, Barthel DW (1965). Functional evaluation: the Barthel Index. Md State Med J.

[17] Katz S (1983). Assessing self-maintenance: activities of daily living, mobility, and instrumental activities of daily living. J Am Geriatr Soc.

[18] Lawton MP, Brody EM (1969). Assessment of older people: self-maintaining and instrumental activities of daily living. Gerontologist.

[19] Oort Q (2017). Development of a questionnaire measuring instrumental activities of daily living (IADL) in patients with brain tumors: a pilot study. J Neurooncol.

[20] Keith RA (1987). The functional independence measure: a new tool for rehabilitation. Adv Clin Rehabil.

[21] ICF CHECKLIST Version 2.1a, Clinician form for international classification of functioning, disability and health. World Health Organization, 2003

[22] de Haes JC, van Knippenberg FC, Neijt JP (1990). Measuring psychological and physical distress in cancer patients: structure and application of the Rotterdam Symptom Checklist. Br J Cancer.

[23] Sander AM (1999). The community integration questionnaire revisited: an assessment of factor structure and validity. Arch Phys Med Rehabil.

[24] McPherson KM (1996). An inter-rater reliability study of the Functional Assessment Measure (FIM + FAM). Disabil Rehabil.

[25] Stewart AL, Ware JE (1992) Measuring functioning and well-being: the medical outcomes study approach. Duke University Press, Durham and London. Psycho-Oncology, 1995. 4(2): p. 163–163

[26] Cohen ME, Marino RJ (2000). The tools of disability outcomes research functional status measures. Arch Phys Med Rehabil.

[27] Gosman-Hedstrom G, Svensson E (2000). Parallel reliability of the functional independence measure and the Barthel ADL index. Disabil Rehabil.

[28] Hsueh IP, Lee MM, Hsieh CL (2001). Psychometric characteristics of the Barthel activities of daily living index in stroke patients. J Formos Med Assoc.

[29] Wade DT, Hewer RL (1987). Functional abilities after stroke: measurement, natural history and prognosis. J Neurol Neurosurg Psychiatry.

[30] D’Olhaberriague L (1996). A reappraisal of reliability and validity studies in stroke. Stroke.

[31] Fortinsky RH, Granger CV, Seltzer GB (1981). The use of functional assessment in understanding home care needs. Med Care.

[32] Asberg KH, Nydevik I (1991). Early prognosis of stroke outcome by means of Katz Index of activities of daily living. Scand J Rehabil Med.

[33] Ferretti-Rebustini RE (2015). Validity of the Katz Index to assess activities of daily living by informants in neuropathological studies. Rev Esc Enferm USP.

[34] Katz S (1970). Progress in development of the index of ADL. Gerontologist.

[35] Liu KP (2007). Activities of daily living performance in dementia. Acta Neurol Scand.

[36] Duncan P (1998). A randomized, controlled pilot study of a home-based exercise program for individuals with mild and moderate stroke. Stroke.

[37] Cheng SK, Man DW (2006). Management of impaired self-awareness in persons with traumatic brain injury. Brain Inj.

