# Supplementary material for: Evaluation of the content coverage of questionnaires containing basic and instrumental activities of daily living (ADL) used in adult patients with brain tumors
Source: J Neurooncol. 2019 Mar 18;143(1):1–13. doi: 10.1007/s11060-019-03136-9 (PMC6482128; doi:10.1007/s11060-019-03136-9)
Supplement: Supplementary file 1 — Supplementary material 1 (DOCX 14 KB) [file 11060_2019_3136_MOESM1_ESM.docx]

**Supplementary File. Search string**

**Brain tumor**

(("Glioma"[Mesh] OR glioma*[tiab] OR glio-ma*[tiab] OR gli-oma*[tiab] OR glyoma*[tiab] OR glyo-ma*[tiab] OR glyoma*[tiab] OR neuroglioma*[tiab] OR neuroglyoma*[tiab] OR ((neoplasm*[ti] OR neoplasm*[ti] OR tumor*[ti] OR tumour*[ti] OR cancer*[ti] OR malignan*[ti]) AND (glia*[ti] OR neuroglia*[ti])) OR glial neoplasm*[tiab] OR "glial neo-plasm"[tiab] OR "glial neo-plasms"[tiab] OR glial cell neoplasm*[tiab] OR "glial cell neo-plasm"[tiab] OR "glial cell neo-plasms"[tiab] OR glial brain neoplasm*[tiab] OR "glial brain neo-plasm"[tiab] OR "glial brain neoplasms"[tiab] OR "glial cns neoplasm"[tiab] OR "glial cns neo-plasm"[tiab] OR "glial cns neoplasms"[tiab] OR "glial cns neo-plasms"[tiab] OR glial tumor*[tiab] OR glial tumour*[tiab] OR glial cell tumor*[tiab] OR glial cell tumour*[tiab] OR glial brain tumor*[tiab] OR glial brain tumour*[tiab] OR "glial cns tumor"[tiab] OR "glial cns tumors"[tiab] OR "glial cns tumour"[tiab] OR "glial cns tumours"[tiab] OR glial cancer*[tiab] OR "glial cell cancer"[tiab] OR "glial cell cancers"[tiab] OR "glial brain cancer"[tiab] OR "glial brain cancers"[tiab] OR "glial cns cancer"[tiab] OR "glial cns cancers"[tiab] OR glial malign*[tiab] OR "glial cell malignancy"[tiab] OR "glial cell malignancies"[tiab] OR "glial brain malignancy"[tiab] OR "glial brain malignancies"[tiab] OR "glial cns malignancy"[tiab] OR "glial cns malignancies"[tiab] OR malignant glia*[tiab] OR malignant glial *[tiab] OR xanthoastrocytoma*[tiab] OR xantoastrocytoma*[tiab] OR astrocytoma*[tiab] OR astro-cytoma*[tiab] OR astroglioma*[tiab] OR astro-glioma*[tiab] OR oligoastrocytoma*[tiab] OR oligoastro-cytoma*[tiab] OR glioblastom*[tiab] OR glio-blastom*[tiab] OR oligodendroglioma*[tiab] OR oligodendroglioma*[tiab] OR oligoden-droglioma*[tiab] OR oligodendroblastoma*[tiab] OR oligodendro-blastoma*[tiab] OR oligo-dendroblastoma*[tiab] OR oligoden-droblastoma*[tiab] OR oligodendro-blastoma*[tiab] OR ependymom*[tiab] OR ependimom*[tiab] OR subependymom*[tiab] OR subependimom*[tiab] OR gliosarcoma*[tiab] OR gliosarcoma*[tiab] OR brain malign*[tiab] OR malignant primary brain*[tiab] OR primary malignant brain*[tiab] OR malignant brain*[tiab]) OR ("Brain Neoplasms"[Mesh] OR tumour of brain*[tiab] OR tumor of brain*[tiab] OR "tumours of brain"[tiab] OR "tumors of brain"[tiab] OR "tumours of brainstem"[tiab] OR "tumors of brainstem"[tiab] OR tumour of the brain*[tiab] OR tumor of the brain*[tiab] OR "tumours of the brain"[tiab] OR "tumors of the brain"[tiab] OR "tumours of the brainstem"[tiab] OR "tumors of the brainstem"[tiab] OR brain tumour*[tiab] OR brain tumor*[tiab] OR brainstem tumour*[tiab] OR brainstem tumor*[tiab] OR brain-stem tumour*[tiab] OR brain-stem tumor*[tiab] OR frontal tumour*[tiab] OR frontal tumor*[tiab] OR "tumour of cns"[tiab] OR tumor of cns*[tiab] OR "tumours of cns"[tiab] OR "tumors of cns"[tiab] OR tumour of the cns*[tiab] OR tumor of the cns*[tiab] OR "tumours of the cns"[tiab] OR "tumors of the cns"[tiab] OR cns tumour*[tiab] OR cns tumor*[tiab] OR "tumour of central nervous system"[tiab] OR tumor of central nervous system*[tiab] OR "tumours of central nervous system"[tiab] OR "tumors of central nervous system"[tiab] OR tumour of the central nervous system*[tiab] OR tumor of the central nervous system*[tiab] OR "tumours of the central nervous system"[tiab] OR "tumors of the central nervous system"[tiab] OR central nervous system tumour*[tiab] OR central nervous system tumor*[tiab] OR intracranial tumour*[tiab] OR intracranial tumor*[tiab] OR intra-cranial tumour*[tiab] OR intra-cranial tumor*[tiab] OR cerebral tumour*[tiab] OR cerebral tumor*[tiab] OR intracerebral tumour*[tiab] OR intracerebral tumor*[tiab] OR neoplasm of brain*[tiab] OR "neoplasms of brain"[tiab] OR "neoplasms of brainstem"[tiab] OR neoplasm of the brain*[tiab] OR "neoplasms of the brain"[tiab] OR "neoplasms of the brainstem"[tiab] OR brain neoplasm*[tiab] OR brainstem neoplasm*[tiab] OR brain-stem neoplasm*[tiab] OR frontalneoplasm*[tiab] OR neoplasm of cns*[tiab] OR "neoplasms of cns"[tiab] OR neoplasm of the cns*[tiab] OR "neoplasms of the cns"[tiab] OR cns neoplasm*[tiab] OR "neoplasm of central nervous system"[tiab] OR "neoplasms of central nervous system"[tiab] OR neoplasm of the central nervous system*[tiab] OR "neoplasms of the central nervous system"[tiab] OR central nervous system neoplasm*[tiab] OR intracranial neoplasm*[tiab] OR intra-cranial neoplasm*[tiab] OR cerebral neoplasm*[tiab] OR intracerebral neoplasm*[tiab] OR cancer of brain*[tiab] OR "cancers of brain"[tiab] OR "cancers of brainstem"[tiab] OR cancer of the brain*[tiab] OR "cancers of the brain"[tiab] OR "cancers of the brainstem"[tiab] OR brain cancer*[tiab] OR "brainstem cancer"[tiab] OR "brainstem cancers"[tiab] OR "brain-stem cancer"[tiab] OR "brain-stem cancers"[tiab] OR "frontal cancer"[tiab] OR "frontal cancers"[tiab] OR "cancer of cns"[tiab] OR "cancers of cns"[tiab] OR "cancer of the cns"[tiab] OR "cancers of the cns"[tiab] OR cns cancer*[tiab] OR "cancer of central nervous system"[tiab] OR "cancers of central nervous system"[tiab] OR cancer of the central nervous system*[tiab] OR "cancers of the central nervous system"[tiab] OR central nervous system cancer*[tiab] OR intracranial cancer*[tiab] OR "intra-cranial cancer"[tiab] OR "intra-cranial cancers"[tiab] OR cerebral cancer*[tiab] OR "intracerebral cancer"[tiab] OR "intracerebral cancers"[tiab] OR "carcinoma of brain"[tiab] OR "carcinomas of brain"[tiab] OR "carcinoma of brainstem"[tiab] OR "carcinomas of brainstem"[tiab] OR "carcinoma of the brain"[tiab] OR "carcinomas of the brain"[tiab] OR "carcinoma of the brainstem"[tiab] OR "carcinomas of the brainstem"[tiab] OR brain carcinoma*[tiab] OR "brainstem carcinoma"[tiab] OR "brainstem carcinomas"[tiab] OR "brain-stem carcinoma"[tiab] OR "brain-stem carcinomas"[tiab] OR "frontal carcinoma"[tiab] OR "frontal carcinomas"[tiab] OR "carcinoma of cns"[tiab] OR "carcinomas of cns"[tiab] OR "carcinoma of the cns"[tiab] OR "carcinomas of the cns"[tiab] OR "cns carcinoma"[tiab] OR "cns carcinomas"[tiab] OR "carcinoma of central nervous system"[tiab] OR "carcinomas of central nervous system"[tiab] OR "carcinoma of the central nervous system"[tiab] OR "carcinomas of the central nervous system"[tiab] OR "central nervous system carcinoma"[tiab] OR "central nervous system carcinomas"[tiab] OR "intracranial carcinoma"[tiab] OR "intracranial carcinomas"[tiab] OR "intra-cranial carcinoma"[tiab] OR "intra-cranial carcinomas"[tiab] OR cerebral carcinoma*[tiab] OR "intracerebral carcinoma"[tiab] OR "intracerebral carcinomas"[tiab] OR "malignancy of brain"[tiab] OR "malignancies of brain"[tiab] OR "malignancy of brainstem"[tiab] OR "malignancies of brainstem"[tiab] OR "malignancy of the brain"[tiab] OR "malignancies of the brain"[tiab] OR "malignancy of the brainstem"[tiab] OR "malignancies of the brainstem"[tiab] OR brain malignan*[tiab] OR "brainstem malignancy"[tiab] OR "brainstem malignancies"[tiab] OR "brain-stem malignancy"[tiab] OR "brain-stem malignancies"[tiab] OR "frontal malignancy"[tiab] OR "frontal malignancies"[tiab] OR "frontal malignant"[tiab] OR "malignancy of cns"[tiab] OR "malignancies of cns"[tiab] OR "malignancy of the cns"[tiab] OR "malignancies of the cns"[tiab] OR cns malignan*[tiab] OR "malignancies of central nervous system"[tiab] OR "malignancy of central nervous system"[tiab] OR "malignancy of the central nervous system"[tiab] OR "malignancies of the central nervous system"[tiab] OR central nervous system malignan*[tiab] OR intracranial malignan*[tiab] OR intra-cranial malignan*[tiab] OR cerebral malignan*[tiab] OR intracerebral malignan*[tiab]))

**ADL**:

"Activities of Daily Living"[Mesh] OR adl[tiab] OR activities of daily living*[tiab] OR daily living activit*[tiab] OR limitation of activit*[tiab] OR independent living*[tiab] OR iadl*[tiab] OR everyday function*[tiab] OR functional abilit*[tiab] OR daily function*[tiab]

**Questionnaires**:

"Questionnaires"[Mesh] OR questionnaire*[tiab] OR instrument*[tiab] OR measure*[tiab]
